# A review of geospatial exposure models and approaches for health data integration

**DOI:** 10.1038/s41370-024-00712-8

**Published:** 2024-09-06

**Authors:** Lara P. Clark, Daniel Zilber, Charles Schmitt, David C. Fargo, David M. Reif, Alison A. Motsinger-Reif, Kyle P. Messier

**Affiliations:** 1 National Institute of Environmental Health Sciences, Office of the Scientific Director, Office of Data Science, Durham, NC USA; 2 National Institute of Environmental Health Sciences, Division of Translational Toxicology, Predictive Toxicology Branch, Durham, NC USA; 3 National Institute of Environmental Health Sciences, Office of the Director, Office of Environmental Science Cyberinfrastructure, Durham, NC USA; 4 National Institute of Environmental Health Sciences, Division of Intramural Research, Biostatistics and Computational Biology Branch, Durham, NC USA

**Keywords:** Exposome, Environmental public health, Exposure modeling, Spatiotemporal, Toxicology, Linkage

## Abstract

**Background:**

Geospatial methods are common in environmental exposure assessments and increasingly integrated with health data to generate comprehensive models of environmental impacts on public health.

**Objective:**

Our objective is to review geospatial exposure models and approaches for health data integration in environmental health applications.

**Methods:**

We conduct a literature review and synthesis.

**Results:**

First, we discuss key concepts and terminology for geospatial exposure data and models. Second, we provide an overview of workflows in geospatial exposure model development and health data integration. Third, we review modeling approaches, including proximity-based, statistical, and mechanistic approaches, across diverse exposure types, such as air quality, water quality, climate, and socioeconomic factors. For each model type, we provide descriptions, general equations, and example applications for environmental exposure assessment. Fourth, we discuss the approaches used to integrate geospatial exposure data and health data, such as methods to link data sources with disparate spatial and temporal scales. Fifth, we describe the landscape of open-source tools supporting these workflows.

## Introduction

Geospatial models are a class of statistical and deterministic methods that account for spatial relationships and/or spatiotemporal correlation in the fixed effects (e.g., covariates, mechanisms) and/or random effects (e.g., error terms). These models have been extensively used in many scientific and engineering disciplines such as forestry [1], mining [2, 3], geology [4], and soil science [5, 6]. More recently, geospatial models have been used in environmental and public health for air and water quality [7–10] exposure assessments.

The geospatial models used in exposure assessment vary significantly in terms of physical properties, statistical methods, implementation requirements, and overall accuracy and precision of predictions. However, the vast literature makes comparisons of these exposure methods and health applications difficult. For example, specific technical assessments have been conducted including Hoek et al. [11], which reviewed land-use regression for geospatial exposure assessment applications. Further, VoPham et al. [12] discussed advanced machine learning (ML) and artificial intelligence (AI) approaches for geospatial exposure assessment. Nieuwenhuijsen [13] provided a book of exposure assessment methods in environmental health including chapters on measurement and modeling via geographic information systems and atmospheric dispersion modeling.

These challenges are becoming increasingly important as comprehensive and complex exposure metrics are needed to understand health outcomes. Whereas traditional environmental health studies exposures and health outcomes one chemical at a time, modern environmental health includes the *exposome*, which attempts to understand the totality of exposures across environmental, social, lifestyle, and ecological factors on human health [14]. The primary approach for quantifying the exposome is through advanced analytical chemistry techniques like non-targeted mass spectrometry, which can potentially elucidate novel and unknown chemicals in individuals [15]; however, it still currently suffers from low throughput, high cost, difficult reproducibility, and potential low precision in quantification. Geospatial approaches offer a tractable and complementary approach to quantify the exposome—particularly the external components such as the social and physical-chemical exposome. Therefore, there is a need for a review and vetted resources on approaches for geospatial exposure modeling and linkages to health data.

Applying geospatial models to help quantify the complete exposome involves substantial data engineering challenges. These include integrating large and diverse data streams (e.g., from sensors, models, surveys, and/or electronic health records), with disparate spatial and temporal coverage and scale, while protecting privacy in personal health information. Additionally, such geospatial exposure methods are rapidly evolving, due to advancements in data sources (such as satellite-based sensors), modeling methods (such as machine learning models), and tools (such as software for handling large geospatial datasets). These data challenges and methods span multiple disciplines, from geographic information science to bioinformatics.

Our objective is to provide a technical introduction to and a compendium of geospatial exposure modeling methods through reviewing information that is otherwise scattered across a vast literature. Specifically, we review: (1) key concepts and terminology for geospatial exposure data and modeling, (2) workflows in geospatial exposure model development and health data integration, (3) types of geospatial exposure models used to assess the complete external exposome, including environmental, climate, and social determinants of health, (4) methods to integrate geospatial exposure data with health data, and (5) open-source tools supporting these workflows.

## Background

This section discusses key terminology and concepts for geospatial exposure data and modeling.

### Terminology

Here, we outline the terminology and notation used throughout the manuscript. Most approaches are statistical or stochastic by definition, so we assume corresponding notation. We briefly mention mechanistic model notation, which is discussed in section “Mechanistic or chemical transport”. A single random variable is denoted by a bold lowercase letter, **y**. A collection of random variables across a spatial and/or temporal domain is denoted as a bold capital letter, **Y**. In the geospatial context, **Y** provides a full, probabilistic characterization of an exposure across space and time and is typically called a space/time random field (S/TRF). An S/TRF is referenced to a real-valued domain with spatial and temporal indices, $\{{{\bf{Y}}}(s,t);s\in {{\mathbb{R}}}^{2},t\in {{\mathbb{R}}}^{1}\}$, where the spatial dimension could be 1 or more than 2. For brevity in equations, let a single spatiotemporal index, *p* = (*s*, *t*), be equal to the combined spatial and temporal index, $\{{{\bf{Y}}}(p);p\in {{\mathbb{R}}}^{3}\}$.

When we describe the relationship between two spatiotemporal observations, parameter estimates, or model predictions, we can utilize generic subscripts, _*ij*_. As is standard in matrix notation, let the first subscript represent row indices and the second represent column indices (and so on for higher dimensions), thus *A*_*ij*_ is the *i*-th row and *j*-th column of *A*.

Full quantification of an S/TRF is the goal of a geospatial exposure assessment; however, the realization is typically noisy. We denote a deterministic value or realization of a random variable of interest with regular, non-bold font, $Y={({y}_{1},...,{y}_{n})}^{T}$. Thus a given realization, *y*_*i*_, can be thought of as a single random draw from an underlying random distribution, **y**_*i*_. Moreover, all statistical models represent a latent, smooth estimation of the exposure of interest:

2.1.1 $${z}_{i}={y}_{i}-{\varepsilon }_{i}$$

where *z*_*i*_ is the latent estimate, *y*_*i*_ is the observed, noisy data, and *ε*_*i*_ is the error. Mechanistic models, or models based on explicit physics and chemistry, do not typically include an error term and thus directly estimate *y*_*i*_.

### Distance metrics

Distance, both spatial and temporal, is the foundation of geospatial exposure metrics, thus it is imperative to define the types of distances. The most common distance is the Euclidean distance, or the “way the crow flies”, a unique value that represents the shortest distance between 2 or more points. Let *A* be a matrix of size [*n* x 2] such as outcome locations or grid locations where an entire exposure field is calculated locations (i.e., x coordinate, y coordinate) and *B* be a [*m* x 2] matrix such as pollution sources. The euclidean distance between *i*-th (*i* = 1, . . . , *n*) object in *A* and the *j*-th (*j* = 1, . . . , *m*) object in *B* is:

2.2.1 $${d}_{ij}=\sqrt{{({A}_{i1}-{B}_{j1})}^{2}+{({A}_{i2}-{B}_{j2})}^{2}}$$

where *d*_*ij*_, a [*n* x *m*] matrix, is the euclidean distance between points all the points in *A* and *B*.

Geodesic distance is used to calculate distances on the Earth’s surface that account for the Earth’s three-dimensional shape, which is typically modeled as an oblate spheroid. When the coordinates are expressed in longitude (x-coordinate) and latitude (y-coordinate), a geographic coordinate system, then a distance calculation must use the geodesic version otherwise the distance calculations can be inaccurate. Euclidean distances are valid in the two-dimensional projections of geographic coordinate systems called projected coordinate systems.

**Fig. 1:**
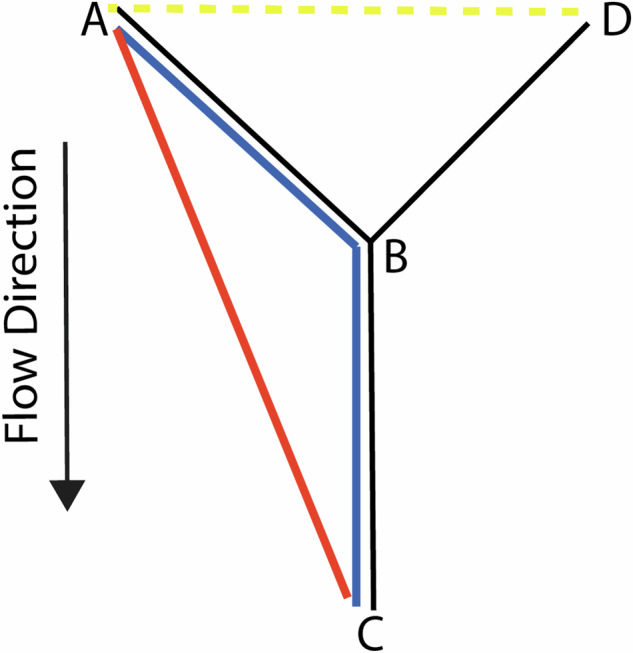
Comparison of distance metrics. The river distance, **A** to **B** to **C** (blue path), is longer than the euclidean distance of **A** to **C** (red path). Moreover, if flow direction is considered, then the river distance from **A** to **D** is infinite (dotted yellow path), since they cannot be connected while constrained to the river network and flow.

In hydrological applications, a non-Euclidean river-distance is often used that is constrained to the path of a given river system. The river distance incorporates the geometry of the river network and potentially flow direction (See Fig. 1), resulting in better accuracy and precision for exposures predominately controlled by river hydrology [16–18]. For the remainder of this review, unless otherwise specified, we refer to the euclidean distance.

Time may be viewed as an additional spatial dimension and modeled on a continuous domain. In this case, the distances may be treated equally (i.e., a distance of 1 unit is the same in space or time) or they may be weighted or scaled based on expert knowledge or model parameters. For example, a combined spatiotemporal distance may be calculated as follows:

2.2.2 $${d}_{\gamma }\left(({{{\bf{s}}}}_{i},{t}_{i}),({{{\bf{s}}}}_{j},{t}_{j})\right)=\sqrt{\frac{| | {{{\bf{s}}}}_{i}-{{{\bf{s}}}}_{j}| {| }_{2}^{2}}{{\gamma }_{s}^{2}}+\frac{{({t}_{i}-{t}_{j})}^{2}}{{\gamma }_{t}^{2}}},$$

where the spatial distance is scaled by a spatial range parameter, *γ*_*s*_, and the temporal distance is scaled by the temporal range parameter, *γ*_*t*_. Range parameters provide an estimate or interpretation to the length or distance at which the process varies.

In dynamic spatial models, time is explicitly considered as a separate and discrete distance. For instance, a joint spatiotemporal process is viewed as a discrete time series of spatial process. For a brief or extensive review of dynamic spatiotemporal distances and models see Wikle [19] and Cressie and Wikle [20], respectively.

## Workflows

This section provides an overview of workflows for environmental health research incorporating geospatial exposure models and a discussion of key steps in geospatial exposure model development.

### Overview

**Fig. 2:**
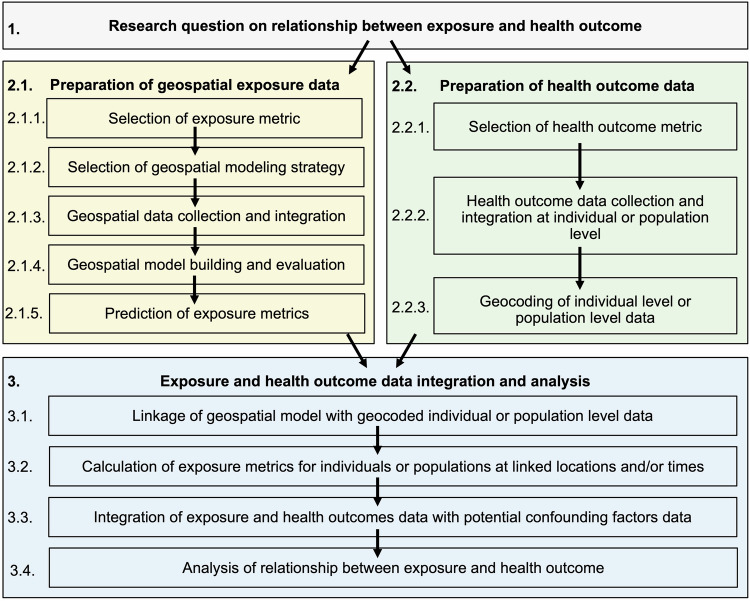
Overview of key steps in environmental health research incorporating geospatial model development for exposure assessment. Key steps include formulation of a research question on the relationship between exposures and health outcomes (panel 1), preparation of geopsatial exposure data (panel 2.1) and health outcome data (panel 2.2), and integration and analysis of the exposure and health outcome data (panel 3).

Figure 2 presents an overview of key steps in environmental health research workflows incorporating geospatial model development for exposure assessment. The first step is formulation of a research question on the association between exposures and health outcomes for specific places (i.e., study area) and times (i.e., study period).

The research question shapes the workflow in several important ways. The study area and period determine the spatial and temporal range (or coverage) of input data needed. The selected exposure and health outcome determine the spatial and temporal scale (or resolution) of the input data needed: finer scale data is needed for exposures and health outcomes with higher spatial and/or temporal variability. The selected health outcome influences the scale of data integration for analysis: input data are typically spatially and temporally integrated to match the health outcome scale (e.g., to home addresses or ZIP codes during specific days or years).

The second step is preparation of the input data with the spatiotemporal range and scale needed to investigate the research question. Preparation of geospatial exposure data through geospatial model development involves the following steps:

- Selection of the exposure metric. This metric quantifies an aspect of the external exposome. Example metrics are average annual air-pollutant concentration and daily maximum heat index.
- Selection of geospatial modeling strategy for the selected exposure metric. This includes selecting the type of model (section “Types of models”), sources of input data, and methods for building and evaluating the model. The spatiotemporal range and scale of analysis are important considerations in selecting the strategy.
- Collection and integration of geospatial data needed as inputs for the selected model. This may involve collecting new measurements (e.g., sampling local air quality) and/or gathering existing measurements from disparate data sources (e.g., accessing global satellite imagery). The input data can include observations of the exposure metric and/or various *geographic covariates* (section “Geographic covariate development”) used to predict the exposure metric at unobserved locations or times. Such geographic covariates are selected based on domain expertise to have an interpretable influence on the exposure metric; they typically represent *sources* of exposure as well as *change* and *transport* processes impacting exposure.
- Building and evaluation of the geospatial model using the selected approaches with the integrated input data. This can involve various model selection and validation methods (section “Spatiotemporal model assessment and selection”).
- Prediction of the exposure metric. This involves applying the geospatial model with input geospatial data to predict the exposure metric at unmonitored space/time locations such as residential addresses or areal units during specific days or years.

Preparation of health outcome data involves the following steps:

- Selection of the health outcome metric. This metric describes a health outcome at the individual level (e.g., for a specific patient or research participant) or at the population level (e.g., among all people living in a specific county). Example metrics are asthma emergency department visits and blood pressure.
- Collection and integration of the health outcome data. This may involve collecting new measurements (e.g., through surveys administered to health cohorts) or gathering information from existing health data sources such as electronic health records, insurance claims, public health surveillance programs, and existing health cohorts. Data catalogs (e.g., the Climate and Health Outcomes Research Data Systems (CHORDS) catalog [21] and others reviewed in section “Open-source tools”) provide examples of health data sources with descriptions of the available data types (e.g., conditions, prescriptions), population characteristics, and privacy-related restrictions on access and use (section “Special health data linkage considerations”).
- Geocoding the health data. This step connects an individual or population to the spatial information needed for linkage to the geospatial exposure data (section “Special health data linkage considerations”). For individual-level data, this may involve geocoding, which is the process of translating addresses (i.e., street address listings) to coordinates (i.e., latitude and longitude). For population-level data, this may involve coding with standard geographic units (e.g., spatial boundaries for counties or postal codes).

The third step is the integration and analysis of the geospatial exposure data and health outcome data. This step involves calculating exposure metrics, using the geospatial exposure model to predict exposures at the specific spatial locations and times linked with each individual or population (section “Geospatial data integration”). Potential confounding factors such as age, social determinants of health, and other types of environmental exposures are then commonly linked with each individual or population. These steps produce an integrated exposure and health dataset. This integrated dataset can then be analyzed using various epidemiological methods to quantify associations between exposures and health while accounting for potential confounding factors.

### Model development

This section discusses key steps in model development workflows.

#### Geographic covariate development

The fundamental drivers of most exposure assessment models are geographic covariates. A design matrix, *X*(*p*), consists of a combination of spatial and/or spatiotemporally referenced geographic covariates. Here, we explain the properties of and broadly define types of covariates that are subsets of the full design matrix. There are two domains of classification to consider in the development of covariates: the *mechanism* that the covariate represents and the *geometry* of the spatial representation.

Geographic covariates represent mechanistic approximations of the outcome of interest and are intended to be interpretable. The mechanisms can be classified into three types of variables, which helps ensure all relevant variables are considered. *Source* variables, *X*^*s*^(*p*), are considered direct or indirect sources of the outcome of interest. For instance, in air pollution studies of NO_2_, internal-combustion vehicles are a direct source since they directly emit NO_2_. *Change* variables, *X*^*c*^(*p*), are processes that may attenuate or concentrate the dependent variable through physical and chemical changes or transformations. For example, ground or surface water nitrate models include soil variables that have properties favorable to denitrification (i.e., decrease) of ${{{\rm{NO}}}}_{3}^{-}$ [22]. In air pollution modeling, solar radiation and urban morphology can inform on transformations such as secondary organics formation, aerosol nucleation, adhesion, and physical constraints such as urban structures. *Transport* variables, *X*^*t*^(*p*), are processes that affect the movement of dependent variables (e.g., advection, diffusion) such as wind or water flow. The distinction of *X*^*c*^(*p*) and *X*^*t*^(*p*) is somewhat arbitrary at this stage; however, it is important for a priori understanding of physical and chemical processes for interpretation and validation. Moreover, many algorithms impose constraints on covariate groups, which enforces physical relevance and can aid in estimation by reducing the overall parameter search space. The geographic covariate design matrix is equal to the set containing the source, change, and transport variables:

3.2.1 $$X(p)=\{{X}^{s}(p),{X}^{c}(p),{X}^{t}(p)\}$$

Source covariates can be categorized as point or non-point sources. Point sources originate at an exact geographic location and are easily represented as a point. These can be calculated with many of the proximity variables from section “Proximity”, such as a nearest distance or the sum of exponentially decaying contribution of sources. Conversely, non-point sources are either unknown in exact origin or occur over a given line or area. In water quality exposure assessment, the impact of agriculture is often described as non-point since large agricultural areas may be a source of pollution, but the source cannot be pinned to a precise geographical location. A common metric is the percentage of a given land cover type such as developed or agricultural land, represented by areal raster or polygon data, within a buffer around the outcome locations. Change and transport variables are also typically derived from areal raster or polygon data. These variables are also best represented with proximity metrics such as percentage of an attenuating land cover type within a buffer (section “Proximity”).

Geographic covariate development is a key step in the exposure assessment model development (Fig. 2). Using a combination of subject expertise and literature reviews, exposure modelers decide which geographic covariates to include in the model. Recommended inclusion characteristics are [23]: (1) Data quality; Data accuracy and precision, strengths, and limitations should be documented and understood. (2) Spatial and temporal scales; Spatial and temporal resolution should be considered within the context of the outcome and the spatial and temporal domains. (3) Geographic and temporal coverage; The domain of the data should include the domain of interest, including prediction locations. (4) Scientific relevance; Covariates should reasonably represent a source, change, or transport process.

The model predictions described in Section “Types of models” are also examples of geographic covariates. For example, proximity models (section “Proximity”) and chemical-transport models (section “Mechanistic or chemical transport”) are common geographic covariates in land-use regression (section “Land-Use Regression”), kriging/Gaussian Process (section “Geostatistical Models: Gaussian Processes, Kriging, and BME”), and machine learning models (section “Machine learning”). Model predictions can serve as covariates or be combined with a post-hoc learning method to produce a hybrid model (section “Hybrid”). Creativity is the only limit to developing helpful and meaningful geographic covariates.

#### Spatiotemporal model estimation

Parameter estimation is an important part of statistical model development regardless of the scientific discipline. The benchmark goal is typically the unbiased estimation of parameters as close to the unobserved “truth” as possible. In exposure modeling, parameter estimation is often important for identifying and quantifying the contribution from sources or reduction from attenuation factors. There are three main approaches for estimating or fitting statistical models: maximum likelihood estimation (MLE), Bayesian inference, and least-squares fitting of empirical estimates. In section “Types of models”, we assume that models are estimated with MLE unless otherwise specified. We recommend Gelfand et al. [24] for in-depth discussions on estimation methods for geospatial models.

#### Spatiotemporal model assessment and selection

**Fig. 3:**
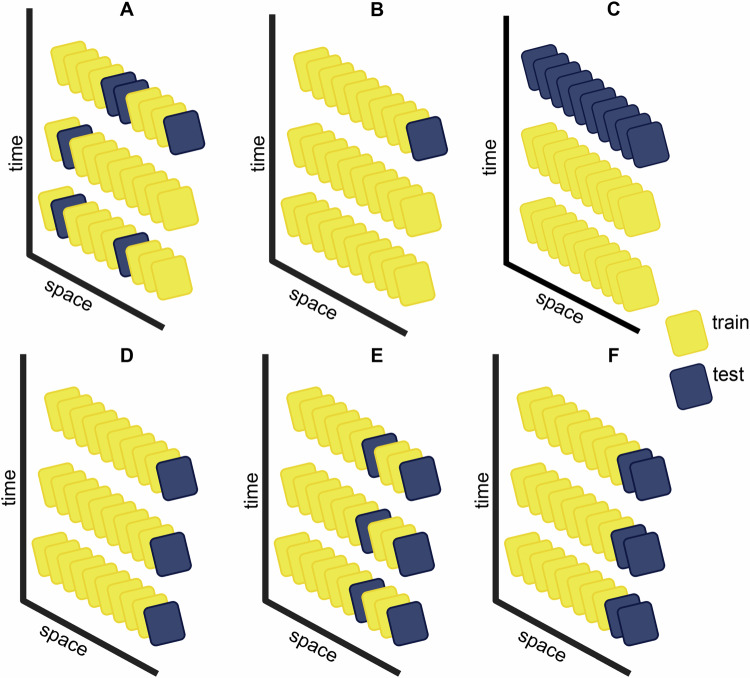
Example cross-validation schemes for spatiotemporal data. **A** Randomly partitioned k-fold cross-validation scheme (i.e., regular k-fold); (**B**) Leave-one-out (LOO); (**C**) Leave-time-out (LTO); (**D**) Leave-location-out (LLO); (**E**) Randomly spaced k-fold leave-location-out (Random LLO); (**F**) Block leave-location-out (Block-LLO). The key difference between random and block LLO, is that the latter folds are all contiguous geographical sets. Blocks can be constructed via a regular square grid, hexagonal grids, clusters, or geographic and political features (e.g., states) [25, 27]. Each square represents a sample in the space-time domain. In each subfigure, the x-axis is the spatial dimension(s), and the y-axis is the time dimension. Thus, there are 10 unique spatial locations with three time points for each location in this example. The spatial spacing is regular (i.e., gridded) for simplicity, but the schemes apply to randomly spaced data. In this example, the test data (blue rectangle) represents one k-fold test set. The complete cross-validation would proceed in a manner such that every training data point (yellow rectangle) eventually serves as a test set exactly once.

In many geospatial exposure applications, prediction at unobserved locations is the primary objective, so the model estimation and selection strategy must reflect this objective. Recently, many authors [25–28] have proposed spatiotemporal-specific cross-validation strategies that account for the spatiotemporal correlation in models and more accurately reflect their out-of-sample or extrapolation prediction capabilities. Figure 3 is a schematic of cross-validation schemes, including versions that produce fairer estimates of prediction errors for spatiotemporal data. Purely random folds (Fig. 3A) and leave-one-out (LOO) (Fig. 3B) cross-validation can result in overly optimistic estimation of the generalization error for spatiotemporal models. Watson et al. [28] recommended a spatial, temporal, or spatiotemporal version based on the space-time sampling strategy and goals of the prediction model. Options include leave-time-out (LTO) (Fig. 3C) or leave-location-out (LLO) (Fig. 3D), which are appropriate for models with sparse temporality or spatial clustering, respectively. Generalizing LLO to random folds (Fig. 3D) or spatially structured blocks (Fig. 3E) results in k-fold and blocked LLO cross-validation, respectively. Roberts et al. [25] and Valavi et al. [27] proposed strategies for developing spatial and temporal blocks based on regular grids, spatial clusters such as k-means, and structures such as watersheds, ecoregions, or political units. Moreover, they proposed the use of spatial buffers, an additional non-active set, between-training and held-out sets. Following Roberts et al. [25] and Valavi et al. [27], we recommend the usage of spatial or temporal block sets where the structure and size are based on correlation in the data and the overall prediction goals. If far-distance extrapolation is not needed, then LLO or LTO is sufficient whereas block LLO may be overly pessimistic [28].

Useful validation statistics for model cross-validation are *R*^2^ and the mean-squared error (MSE). *R*^2^ is bounded between 0 and 1, with larger values indicating a higher proportion of the overall variance described the model. MSE is bounded from 0 to infinity (lower is better) and includes bias, variance, and irreducible error. Gneiting and Katzfuss [29] and many others prior argued for consideration of the joint distribution of predictions and observations. More simply, model assessment and selection methods should consider both prediction point statistics (e.g., mean, median) and the uncertainty quantification (e.g., variance). A proper scoring rule such as the continuous ranked probability score (CRPS) evaluates and penalizes not only the central tendency prediction (e.g., mean) but also under and over confident predictions of uncertainty. For example, an exposure assessment with large variance predictions can claim the predictions never fall outside a given range. CRPS penalizes poor variance estimates, which encourages exposure models with better usage in downstream risk assessment. Moreover, CRPS reduces to the MSE for point predictions. The usage of proper scoring rules in geospatial exposure science is uncommon, but they have been utilized effectively in recent years as probabilistic models are becoming more common [30–34]. For predictive models that produce uncertainty quantification, we highly recommend the usage of proper scoring rules (see (author?) 29) for model assessment and validation.

Typically, a large number of potential covariates are calculated, with the final model determined through the selection of dimension reduction approaches. This avoids using a smaller, but easier to test, set of covariates that miss important covariates and lead to less accurate predictions. The goal is to find, *X*^*r*^ ⊆ *X*, where *X*^*r*^ is a subset of the true or best covariates in the large design matrix, *X*. Alternatively, dimension reduction may be used to estimate a new design matrix, *X*^*d*^, whose rank (i.e., independent columns) is much lower than the full design matrix:

3.2.2 $$rank({X}^{d})\ll rank(X)$$

Here, we describe common model selection and dimension reduction techniques developed in the statistical methodology literature that have been successfully applied in geospatial exposure science.

##### Stepwise and Ad-Hoc stepwise

Forward, backward, and stepwise regression are a family of model selection algorithms with a long history of use in many statistical modeling applications [35]. In stepwise regression, variables are added or subtracted one at a time to the model. At each step, the variable with highest correlation or lowest coefficient p-value that is significant (e.g., *F*-test) is added. Variables previously in the model are tested for significance and may be dropped.

In geographic exposure assessments, a modified stepwise procedure is often used. In the European Study of Cohorts for Air Pollution Effects (ESCAPE) study [36], which has subsequently been adopted by the vast majority of land use regression (LUR) exposure science applications [37–39], LUR models were developed using the following modified procedure: (1) Every covariate is regressed against the outcome in univariable models. (2) The variable that results in the largest increase in *R*^2^ that is also greater than 0.01, is significant at *p* ≤ 0.05, abides by directional constraints determined a priori, does not change the direction of previous variables, and does not increase the *p*-value of previous covariates to greater than 0.05, is added to the model. The directional constraints are determined based on the expected mechanistic interpretation of the covariate. Source variables are expected to increase pollution levels only and are thus constrained to be positive. Change and transport variables can increase or decrease pollution levels, so they are typically not constrained. (3) With a given set of covariates, the process continues, with one variable added at a time until the increase in *R*^2^ is not met. An additional constraint for dealing with distance hyperparameters is often included. If a variable such as forest landcover percentage within a buffer is added, subsequent forest landcover buffer variables are either excluded or forced to have a much different distance hyperparameter (i.e., short- and long-range mechanisms).

Modified stepwise procedures for geospatial exposure assessments are the most frequently used approach for model selection and fitting. Other algorithms include a distance decay regression selection strategy [40] and constrained forward non-linear regression with hyperparameter optimization [41]. While these algorithms have been used for developing exposure assessments, the stepwise family of model selection strategies has many well-known limitations. Stepwise algorithms are known as “greedy” algorithms because they update a model one variable at a time, placing high importance on the local choice of one variable over another [42]. Additionally, the algorithm uses hypothesis tests such as *t*- or *f* − tests that were designed for a small number of model tests and thus are not optimal for multiple test comparisons [43]. Lastly, the algorithm does not scale well for a large number of covariates unless additional steps are taken to reduce the candidate set of variables, which can inflate out-of-sample prediction accuracy. For these reasons, researchers have utilized model selection and reduction methods such as penalization and dimension reduction that have better statistical properties.

##### Penalized regression

Penalization (also known as regularization or shrinkage) is a model-fitting and selection technique that utilizes a constraint (i.e., the penalty) on the model coefficients to shrink coefficients towards zero. The epistemological principle of penalization is that the model introduces bias (i.e., deviates from the minimum variance least-squares estimate) into the model coefficients to reduce the variance in each coefficient estimate. Moreover, the algorithms estimate penalized models along “regularization paths,” which reduces covariate coefficients towards zero in a continuous manner rather than all at once [44, 45]. This democratic version of model selection reduces the impact of a single covariate on the model selection process. Lastly, the penalization approaches effectively perform model selection and coefficient estimate simultaneously, avoiding the multiple hypothesis test violations of stepwise approaches.

A penalization can be written as a constrained optimization problem:

3.2.3 $${\min }_{\beta \in {{\mathbb{R}}}^{p}}\quad \{{(Y-f(X;\beta ))}^{2}\}\quad s.t.\quad P(\beta )\le t$$

in which the sum of squared loss on a generic model with input *X* and parameters *β*, *f*(*X*; *β*), is constrained subject to (*s*. *t*) a function, *P*(⋅), being less than or equal to some value, *t*, and *X* is unit-normal standardized so that the solution does not depend on the scale of the covariates. Penalization approaches are widely used in LUR (section “Land-use regression”), Gaussian process (Section “Geostatistical Models: Gaussian Processes, Kriging, and BME”), and machine learning (Section “Machine Learning”) models. For simplicity, we assume that *f*(*X*; *β*) is a simple linear model. The *L*_2_-norm results in ridge regression, can handle high multicollinearity, and provides stable solutions:

3.2.4 $${\min }_{\beta \in {{\mathbb{R}}}^{p}}\quad \{{(Y-X\beta )}^{2}\}\quad s.t.\quad | | \beta | {| }_{2}^{2}\le t$$

However, ridge regression cannot reduce coefficients to zero due to the geometry of the *L*_2_-norm. Least absolute shrinkage and selection operator (lasso) [46] is a popular penalty that performs simultaneous model selection and fitting which uses *L*_1_-norm as the penalty function:

3.2.5 $${\min }_{\beta \in {{\mathbb{R}}}^{p}}\quad \{{(Y-X\beta )}^{2}\}\quad s.t.\quad | | \beta | {| }_{1}\le t$$

There are many more penalties that can be used to perform model fitting and selection, including the elastic-net [47], a combination of lasso and ridge penalties, and non-concave penalties such as the smoothly clipped absolute deviation [45]. Penalization is often written in the so-called Lagrangian form, which leads to a classical linear regression with the Lagrangian multiplier or penalty, *λ*:

3.2.6 $${\min }_{\beta \in {{\mathbb{R}}}^{p}}\quad \{{(Y-X\beta )}^{2}\}+\lambda P(\beta )$$

Equation (3.2.6) allows for faster and tailored unconstrained optimization algorithms such as Newton–Raphson, Nelder–Mead, or coordinate descent. Penalization approaches have been implemented in geospatial environmental exposure assessment applications [48–50]; however, they remain less common than stepwise and ad-hoc approaches. The authors recommend adoption and use of penalization over stepwise and ad-hoc selection approaches.

##### Dimension reduction

Dimension reduction (DR) is another popular approach for reducing the complexity of the model covariate space. Briefly, DR is a general framework for reducing a highly dimensional covariate space to a representative, low-dimensional subspace. The approaches can be described as either linear or non-linear, where the former are usually more interpretable and the latter can capture non-linearities in complex, high-dimensional data. DR is a large area of statistical and applied research, so we highlight the most common methods used in environmental exposure assessment.

Principal components analysis (PCA) is the most basic and well-known dimension reduction technique [51]. Principal components are linear combinations of a normalized, high-dimensional covariate matrix such that each component maximizes the sample variance. The first principal component has the largest sample variance among all normalized linear combinations. Subsequent principal components maximize the variance and are orthogonal to the previous principal components. PCA has been used in geospatial exposure assessments and is particularly useful in Bayesian model fitting techniques where model selection may be difficult or infeasible. Recently, the partial least squares (PLS) linear DR technique was used for big data land-use regression models of fine particulate matter [52] and nitrogen-dioxide [53]. PLS is similar to PCA; however, it includes a dependent variable, *Y*, and maximizes the covariance between *Y* and a high-dimensional space covariate set [52]. It is preferred over PCA since it accounts for relationships across dependent and independent variables [52].

Non-linear DR employs various techniques such as kernels, manifolds, auto-encoders [54], and other projections to transform and cluster high-dimensional data into low-dimensional subspaces. The methods are diverse and complex, and their descriptions is beyond the scope of this work. While non-linear DR methods have been employed in related fields such as pattern recognition and genomics, their implementation is sparse in geospatial exposure science. The authors are aware of only a few exposure assessment studies that have implemented the non-linear DR methods of uniform manifold approximation and projection [55] as part of a larger machine learning approach for the prediction of PM_2.5_ and PM_10_ [56, 57]. In adjacent scientific fields, auto-encoders are being used for dimension reduction of complex spatiotemporal processes in climate and weather modeling [58, 59].

In practice, DR is used as a pre-processing step to reduce a high-dimensional geographic covariate set into a tractable, low-dimensional covariate set that can then be used in subsequent model estimation and prediction (see Equation (3.2.2)). In linear DR, care and domain knowledge are critical for developing and interpreting reduced dimension covariates, and interpretations are typically not as straightforward as the raw covariates. Non-linear DR is advisable when prediction accuracy is the primary goal and clear interpretations are not necessary, as they typically capture more complexity in a smaller subspace that is not possible with linear methods.

## Types of models

This section describes the diverse landscape of models used in geospatial exposure science. Table 1 provides an overview of the models and their basic functional forms, such as *Y* = *f*(*x*) + *ε*.

**Table 1:** Summary of the model names, general formulation, section number, and equation number.

Model	General Formulation	Section Number	Formula Number
Proximity	*Y*(*p*) = *X*_*i*_(*p*)	4.1	(4.1.1)
Land Use Regression	**Y**(*p*) = *X*(*p*)*β* + *ε*	4.2	(4.2.1)
Geographically Weighted Regression	**Y**(*p*) = *X*(*p*)*β*(*p*) + *ε*	4.3	(4.3.1)
Geostatical Models	**Y**(*p*) = *GP*(*μ*(*p*), *Σ*_*θ*_(*p*, p_*_))	4.4	(4.4.1)
Machine Learning	**Y**(*p*) = *f*(*X*(*p*))	4.5	(4.5.1)
Ensemble Model	${{\bf{Y}}}(p)={\sum }_{m = 1}^{M}{w}_{m}{f}_{m}(X(p)){\sum }^{M}{w}_{m}=1$	4.5.2	(4.5.3)
Boosting	*Y*_*m*_(*p*) = *Y*_*m*−1_(*p*) + *νy*_*m*_(*p*)	4.5.2	(4.5.4)
Mechanistic	$Y(p):= \frac{\partial {C}_{i}}{\partial t}=-{{\boldsymbol{\nabla }}}\cdot \left({{\bf{v}}}{C}_{i}\right)+{{\boldsymbol{\nabla }}}\cdot \left(D{{\boldsymbol{\nabla }}}{C}_{i}\right)+\mathop{\sum }_{j = 1}^{n}{r}_{i,j}+{G}_{i}$	4.6	(4.6.1)

Details of each model are provided in the given section number. Note that the statistical models result in random variables represented by bold letters. Machine learning models can produce deterministic or random variables, so the latter is represented here.

### Proximity

**Fig. 4:**
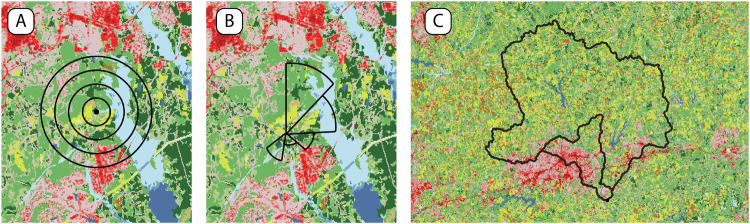
Illustration of common buffer variables applied to land cover classification. **A** Within four isotropic, circular buffers with radii *R*_*j*_, {*j* = 1, . . . , *J*}; (**B**) Within an aniostropic, wind-rose based buffer common in air quality studies; (**C**) Within two upstream contributing areas as commonly in water pollution studies.

Proximity exposure metrics are the most basic form of an exposure assessment because they rely only on the distance between a pollution source and the observed outcome location. Proximity exposure metrics have been used to elucidate the impacts of environmental exposures on human health, including asthma [60], cardiovascular disease [61], and reproductive fertility [62]. From a linear perspective, a proximity model is simply a deterministic covariate:

4.1.1 $$Y(p)=X(p)$$

where *X* is the deterministic quantity calculated at location *p* (note the lack of an error term). Here, we describe the most common and easily calculated proximity metrics. Given a distance matrix, *d*_*ij*_, the minimum distance is:

4.1.2 $${X}_{i}^{min}=\min ({d}_{i,\cdot })$$

where *d*_*i*,⋅_ is the *i*-th row indicating the distance between outcome *i* and every pollution source. The average distance is:

4.1.3 $$\overline{{X}_{i}}=\frac{1}{{n}_{j}}\mathop{\sum }_{j=1}^{{n}_{j}}{d}_{ij}$$

where *n*_*j*_ is the number of pollution sources. Buffer variables are a useful class of proximity metrics. Buffer variables can be calculated for areal, point, and line sources. Summary statistics such as the mean or fraction within a given area around the location of interest can be calculated. Figure 4 illustrates the most common buffer variables.

A proximity metric that incorporates the distance, density, and potential emissions is the sum of the exponentially decaying contribution of point sources, [37]:

4.1.4 $${X}_{i}={\sum }_{j=1}^{J}{C}_{0j}\exp \left(\!-\frac{{d}_{ij}}{r}\right)$$

where *X*_*i*_ is the quantity at location *i*, *C*_0*j*_ is an initial value such as concentration or emissions at source *j*, *d*_*ij*_ is the distance between site *i* and source *j*, *r* is the exponential decay range, and *J* is the number of sources.

Proximity metrics provide a simple exposure assessment model for point, line, and grid data. From an exposure model validation perspective, however, they are limited because there is no observed data to validate the model. For epidemiological studies, proximity metrics are tested directly against health outcome data with model selection or evaluation of multiple models [63]. If monitoring data are available for an exposure of interest, such as a chemical exposure, developing a model that directly predicts the chemical concentration is recommended. Proximity metrics are routinely developed and applied as geographic covariates in other types of exposure models, such as land use regression models.

### Land-use regression

Briggs et al. [64] is largely credited with developing land-use regression (LUR) as a method for estimating air pollution exposure. Coincidentally, a similar method for estimating nutrient loads in river reaches was also introduced in the same year [65]. Land-use regression is simply a linear or nonlinear regression model with spatially-referenced, geographic covariates. The most common linear LUR is:

4.2.1 $${{\bf{Y}}}(p)=X(p)\beta +\varepsilon$$

where **Y**(*p*) are the *n* × 1 observations for the variable of interest (e.g., PM${}_{2.5},N{O}_{3}^{-}$) with space-time locations *s* and *t*, *X*(*p*) is an *n* × *k* design matrix of *k* spatial and/or spatiotemporal geographic covariates, *β* is a *k* × 1 vector of linear regression coefficients, and *ε* is the *n* × 1 vector of independent and identically distributed (i.i.d.) errors typical of a classical linear regression. Surface water [65] and similar groundwater models [22, 41] use nonlinear regression with linear source terms multiplied by exponential attenuation and transport terms.

A strength of LUR models is their flexibility to include other models and data sources as interpretable geographic covariates. The exposure models discussed in subsequent sections can be included as covariates in LUR models. Another strength of LUR geographic covariates is their flexibility with respect to distance parameters (i.e., distance hyperparameter). The distance hyperparameter is typically unknown, and thus calculating many of the same variables with varying distance hyperparameters is recommended [37, 40]. Model selection or dimension reduction is used to determine the best covariates and corresponding distance hyperparameters and provide insight into the spatial and temporal scales of the process of interest.

LUR models can be used to make exposure predictions at any location where geographic covariates exist. LUR model prediction mean and variance follow the same formulation as standard linear regression models. The prediction mean, $\hat{Y}$, at new location, p_*_, is:

4.2.2 $$\hat{Y}({p}_{* })=X({p}_{* })\hat{\beta }$$

where $\hat{\beta }$ is the estimated coefficient vector in equation (4.2.1). The LUR prediction variance at a new location, p_*_, depends on the variation in the residuals and the variation of estimating the true mean with the predictions [66]. It follows that the prediction variance is:

4.2.3 $$Var(\hat{Y}({p}_{* }))=Var(X({p}_{* })\hat{\beta })+Var(\varepsilon )$$

The variance of the residuals is the sample variance, ${S}_{Y}^{2}$. Expanding each component of equation (4.2.3), the general formulation can be written as [66]:

4.2.4 $$Var(\hat{Y}({p}_{* }))={S}_{Y}^{2}[1+X({p}_{* }){[X{(p)}^{T}X(p)]}^{-1}X{({p}_{* })}^{T}]$$

This general formulation for prediction variance is also seen in other regression-based models (Sections “Geographically Weighted Regression” and “Geostatistical Models: Gaussian Processes, Kriging, and BME”).

### Geographically weighted regression

Geographically weighted regression (GWR) is an extension of linear regression and LUR models that allows for spatially and/or spatiotemporally varying coefficients [67]. The main principal behind GWR is that the coefficients in spatial models are non-stationary; that is, the properties or values of model parameters vary depending on local conditions. The GWR extension of linear regression can be written as [68]:

4.3.1 $${{\bf{Y}}}(p)=X(p)\beta (p)+\varepsilon$$

where *Y*(*p*), *X*(*p*), and *ε* are the spatiotemporally varying outcome, spatiotemporal covariate, and i.i.d. error and are the same as in equation (4.2.1). *β*(*p*) are now spatially and temporally referenced. Mathematically, the coefficient estimates can be considered a version of generalized least squares [68] or a random effect model [69]. For the former, the coefficients are:

4.3.2 $$\beta (p)={\left(X{(p)}^{T}W{(p)}^{-1}X(p)\right)}^{-1}X{(p)}^{T}W{(p)}^{-1}Y(p)$$

where *W*(*p*) is a spatiotemporal weight matrix. Gelfand et al. [69] provide the general specification of the random effects approach for spatially varying coefficients as:

4.3.3 $$\tilde{{\beta }_{k}}(p)={\beta }_{k}+{\beta }_{k}(p)$$

which can interpreted as a spatially-varying random adjustment, *β*_*k*_(*p*), at locations *p* to the overall slope *β*_*k*_.

While GWR is not as popular as LUR in environmental exposure assessment, it is gaining favor and has been successfully implemented in a few cases. Hu et al. [70] and Van Donkelaar et al. [71] both implemented a GWR for a PM_2.5_ model that integrated a variety of geospatial covariates. van Donkelaar et al. [72] utilized GWR to estimate PM_2.5_ chemical composition, such as nitrates, sulfate, and organic matter. Kloog et al. [73] and Kloog et al. [74] developed random-effects models for prediction of PM_2.5_.

Brunsdon et al. [67] and Fotheringham et al. [68] provided frameworks for estimating the GLS style GWR models, including a spatiotemporally varying weight matrix, *W*(*p*). Briefly, choices on bandwidth or the distance at which coefficients are smoothed, must be made. This includes choices on the bandwidth distance and the smoothing function (e.g., inverse-distance, Gaussian kernel). Algorithms are available to estimate these parameters systematically through a cross-validation procedure; however, more flexibility in estimation is directly related to increased computational burden. To simplify choices and computation time, Van Donkelaar et al. [71] weight coefficient estimates according to an inverse-distance from observations. Gelfand et al. [69] provide the details such as the likelihood derivations and Bayesian estimation approaches for the spatially-varying random effects approach including the posterior predictives estimates.

GLS-style GWR models also have a straightforward approach for making exposure predictions at any location where the geographic covariates exist. GWR model prediction mean and variance are similar to LUR, but with modifications for the spatially varying coefficients and weights matrix. The prediction mean, $\hat{Y}$, at new location, p_*_, is:

4.3.4 $$\hat{Y}({p}_{* })=X({p}_{* })\hat{\beta }({p}_{* })$$

where $\hat{\beta }({p}_{* })$ is the estimated coefficient vector in equation (4.3.1) at location p_*_ computed with (4.3.3). The GWR prediction variance also follows the general formulation of equation (4.2.4). The prediction variance for GWR, which accounts for both estimation of the mean uncertainty and point prediction uncertainty is [75]:

4.3.5 $$Var(\hat{{Y}_{\!\!* }})={S}_{Y}^{2}\left[1+{X}_{\!* }{[{X}^{T}{W}_{\!* }X]}^{-1}[{X}^{T}{W}_{\!* }^{2}X]{[{X}^{T}{W}_{\!* }X]}^{-1}{X}_{\!* }^{T}\right]$$

where the spatiotemporal index, (${{p}_{\!* }}$), is implied for brevity (i.e., $\hat{Y}({p}_{* })$ in equation (4.3.4) is equivalent to $\hat{{Y}_{\!* }}$ in equation (4.3.5)).

### Geostatistical models: Gaussian Processes, Kriging, and BME

Geostatistical models are models that contain explicit error terms to model spatial, temporal, or spatiotemporal auto-correlation in the data. In other words, they are a function to interpolate, extrapolate, or smooth the dependent variable. They have a rich history across many scientific and computational fields including forestry [1], geology [3], engineering [76], statistics [4, 24], machine learning [77] and most recently in environmental health [78]. For this reason, there is often confusion in terminology as nominally equivalent methods were developed in parallel among siloed disciplines. For example, the term “Kriging” is most popular in the engineering and public health literature whereas “Gaussian Process” is more often used in the spatial statistics and machine learning literature.

By definition, a Gaussian process (GP) is a collection of random variables, a finite number of which have a joint Gaussian distribution [77]. A GP is defined by a mean, *μ*(*p*), and covariance between locations, *Σ*(*p*, p_*_)

4.4.1 $${{\bf{Y}}}(p)=GP(\mu (p),{\Sigma }_{\theta }(p,{p}_{* }))$$

Each location is defined by the marginal Gaussian distribution, and thus the number of parameters in the model increases along with an increase in sample size. Hence, *GP* theoretically has an infinite parameter space and is considered non-parametric. *Σ*_*θ*_ is a covariance matrix that is modeled with kernel functions with parameters *θ*.

Geostatistical models can also be written as a mixed-effect model where the covariance between points is contained in the random effects term:

4.4.2 $${{\bf{Y}}}(p)=\mu (p)+\eta (p).$$

*μ*(*p*) can take many forms such as linear, nonlinear, or even ML models such as random forest [79]. Here, *μ*(*p*) is the form of a simple linear model, *Xβ*, and *η*(*p*) is an error term, which can decomposed into independent and identically distributed error and spatiotemporally correlated error represented as a GP, *η* ~ *GP*(0, *Σ*_*θ*_ + *τ*^2^*I*). *Σ*_*θ*_ is a covariance matrix with parameters, *θ*, that accounts for correlation between spatial and temporal locations. Given that **y**_*i*_ has a Gaussian distribution, the vector of space-time observations, **Y**, has a multivariate Gaussian distribution. Thus, we can utilize the probability distribution function of a multivariate Gaussian density to define the likelihood of equation (4.4.2) as [24]:

4.4.3 $$L(\beta ,\theta ;{{\bf{Y}}})={(2\pi )}^{-n/2}| {\Sigma }_{\theta }{| }^{-1/2}\exp \{-{({{\bf{Y}}}-X\beta )}^{T}{\Sigma }_{\theta }^{-1}({{\bf{Y}}}-X\beta )/2\}$$

where ∣*Σ*_*θ*_∣ is the determinant of the covariance matrix, a positive-definite matrix parameterized by the covariance or kernel function. The choice of covariance or kernel functions is an active area of research, but, in exposure science, stationary, symmetric kernel functions such as exponential, Gaussian (squared-exponential), and Matérn are the most common and are recommended. The squared exponential or Gaussian covariance with variance *σ*^2^, length scale (i.e., decay range), parameter *r*, and distance between locations, *d*, is one of the simplest and most common choices:

4.4.4 $$K(d| {\sigma }^{2},r)={\sigma }^{2}exp(-{d}^{2}/r)$$

Letting *σ* = 1, note that as *d* moves toward 0, the correlation moves toward 1: this implies that the covariance and correlation between locations increases as points are closer together, with the rate and overall distance determined by the estimated covariance parameters. The squared exponential also has a special property that ensures functions will be very smooth, or infinitely differentiable.

The Matérn kernel is a generalization of the Gaussian function that introduces a smoothness parameter to control how many times the sample paths can be differentiated. As the smoothness parameter approaches infinity, the squared exponential covariance is recovered. Natural phenomenon tend to have finite differentiability as opposed to infinite differentiability, and the theoretical properties are good [80], so the Matérn covariance is considered an appropriate choice for exposure and health applications.

Bayesian maximum entropy (BME) is a popular geostatistical approach that can be considered an extension of classical geostatistics methods. Like classical geostatistical methods, covariance parameters are estimated based on an empirical estimation of covariance and the approach does not utilize distributional assumptions. The key aspect differentiating BME from classical geostatistics methods is that predictions can include non-Gaussian uncertainty in predictions at new locations. He and Kolovos [81] extensively reviewed BME, including its successful applications in geospatial exposure modeling.

MLE and Bayesian estimation can simultaneously estimate the mean and variance components of equation (4.4.1), which is statistically more optimal than the empirical approach but introduces computational challenges. With a large number of locations, the likelihood, as in equation (4.4.3) becomes computationally difficult to evaluate because the inverse of the covariance matrix *Σ* is dense. Nearest neighbor approximations as predictive processes [82] and general Vecchia approximations [83] are among the simplest and most effective techniques: correlations between points that are far away from each other are essentially ignored. Nearest neighbor approximations are suitable for MLE or Bayesian inference. A popular approach for efficient Bayesian inference is the integrated nested Laplace approximation (INLA), which uses a stochastic partial differential approximation of a multivariate Gaussian random field and the Laplace approximation for posterior distributions [84]. Moran and Wheeler [85] developed a Gibbs sampling algorithm for rapid Bayesian inference utilizing hierarchical matrix approximations. Combined mean and GP estimation is further discussed in section “Hybrid” since the distinction between some GP and hybrid methods is blurry.

Kriging is often referred to as the explicit step using geostatistical models for prediction at new locations. For the covariance matrix *Σ*, we notate the dimension representing new predictions locations with subscript * and observations otherwise. The Kriging prediction mean, $\hat{Y}$, assuming a linear mean, is:

4.4.5 $$\hat{Y}({p}_{* })=X({p}_{* })\hat{\beta }+\left[K({p}_{* },p){[K(p,p)+{\tau }^{2}I]}^{-1}[Y(p)-X(p)\hat{\beta }]\right]$$

where $X({p}_{* })\hat{\beta }$ is the linear mean at prediction locations, *K*(p_*_, *p*) (e.g., Equation (4.4.4)) is the estimated covariance matrix between prediction locations and observations, *K*(*p*, *p*) is the estimated covariance matrix between observations, *τ*^2^*I* is the independent error added to the *K*(*p*, *p*) diagonal, and [$Y(p)-X(p)\hat{\beta }$] is the residual at the observations. The Kriging prediction mean can be described as the linear regression model mean at prediction locations plus the interpolated residuals of the exposure data observations. The strength of the residual interpolation is based on the covariance parameters. The Kriging/GP prediction variance is the following:

4.4.6 $$Var(\hat{Y}({p}_{* }))=K({p}_{* },{p}_{* })-K({p}_{* },p){[K({p}_{,}p)+{\tau }^{2}]}^{-1}K(p,{p}_{* })$$

where *K*(p_*_, p_*_) is the covariance matrix between prediction locations, and $K(p,{p}_{* })=K{({p}_{* },p)}^{T}$ is the transpose of the covariance between prediction locations and observations. The Kriging/GP variance can be described as the total estimated variance at the prediction locations minus the variance from the additional information contributed by the observation residual interpolations.

### Machine learning

Machine learning (ML) describes predictive modeling focused on a learning algorithm and out-of-sample prediction generalization [86]. ML methods have fewer assumptions and are highly parameterized and thus more flexible for capturing complex non-linearity. ML methods for geospatial exposure assessment utilize the same geographic covariates as predictor variables such as LUR (section “Land-use regression”), GWR (section “Geographically weighted regression”), and geostatistical models (section “Geostatistical Models: Gaussian Processes, Kriging, and BME”) and can capture non-linear relationships within and across covariates. Nonetheless, great care is needed to estimate a ML model properly as they are often susceptible to over-fitting. Their success in a wide variety of computational applications has led to their adoption in geospatial exposure modeling. A general ML equation is [87]:

4.5.1 $${{\bf{Y}}}(p)=f(X(p))$$

where *f*( ⋅ ) is a difficult-to-express function and encompasses a wide variety of forms in ML. Prediction for ML models varies, but, in general, ML prediction utilizes parameters from the estimation process and geographic covariates at prediction locations:

4.5.2 $$\hat{Y}({p}_{* })=f(X({p}_{* }))$$

ML models typically estimate a central tendency and do not have an explicit prediction variance equation; however, bootstrapping techniques provide a straightforward way to determine approximate prediction variance. For details on the methodologies of ML models and algorithms including generalized additive models, tree methods, boosted and additive trees, support vector machines, and neural networks, we refer readers to Hastie et al. [88]. Additionally, Yan [87] summarized and provided examples of ML for chemical safety applications, including geospatial exposure assessments. Here, we discuss the basic equations and properties of two classes of ML methods that have been successfully applied to geospatial exposure modeling: neural networks and ensemble models.

#### Neural networks

Neural networks, or artificial neural networks (ANN), are inspired by the structure and function of the human brain. Neural networks consist of layers of interconnected nodes, known as artificial neurons, that process and transmit information through the network. The connections between neurons are weighted, and the weights are updated during the training process to improve the accuracy of the network’s predictions. At their simplest, ANN are essentially repeated logistic regression models. However, ANN represent a modern frontier in statistics and ML where improvements and new models abound. More complicated ANN, referred to as deep learning, allows computational models that are composed of multiple processing layers to learn representations of data with multiple levels of non-linearity and abstraction. For comprehensive explanations of neural networks and deep learning, we refer the readers to Bishop [89], LeCun et al. [90], and Goodfellow et al. [91]. Additionally, Yan [87] provided an overview of ANN in exposure science applications.

ANN have been successfully applied in geospatial exposure modeling. Di et al. [92] and Di et al. [93] developed highly accurate, annual average PM_2.5_ and ozone predictions, respectively. They noted that convolutional layers have the attractive property of accounting for spatial autocorrelation and spatial scale hierarchies (i.e., long range vs. short range). Pyo et al. [94] used ANN to predict cyanobacteria algae blooms in surface water, which are important for human and ecological health applications. Müller et al. [95] and Azimi et al. [96] used ANN to predict groundwater quantity and quality, respectively. ANN have improved predictions of social determinants and their associations with health outcomes [97]. Lastly, Weichenthal et al. [98] discussed future research directions for ANN in exposure science applications.

#### Ensemble methods

Individual geospatial exposure models, no matter how sophisticated, have strengths and weaknesses compared to alternative model choices. For example, one model may capture low concentrations better than high concentrations while another may capture regional variability better than fine, local-scale variability. Ensemble models are a class of ML algorithms based on the simple concept that a large committee of models is better than an individual model. Here, we describe ML methods based on an ensemble of base or weak models. This differs from ensemble models that are combinations of multiple other full models, often referred to as meta-learners, super-learners, or hybrid models. These are discussed in section “Hybrid”.

In an ensemble model, the final prediction is a weighted average of multiple models:

4.5.3 $$Y(p)={\sum }_{m=1}^{M}{w}_{m}{f}_{m}(X(p)),{\sum}^{M}{w}_{m}=1$$

where *f*_*m*_ is an individual model, *m* = 1, …, *M*, and the weights, *w*_*m*_, sum to 1 and are typically estimated through an optimization procedure. If the weights are equivalent, *w*_*m*_ = 1/*M*, then a simple average of base models can be used. If a weighted average is desired, a simple linear model or GAM can serve as the meta-learner.

Hastie et al. [88] extensively explained tree-based ensembles for regression and classification. Briefly, a tree-based model splits data into hierarchical subsets based on certain features and at each split, applies a decision rule to partition the data and fit a simple model such as a constant. The prediction for a new sample is determined by traversing the tree, applying the decision rules at each node until a terminal node is reached, and using the average target value associated with that terminal node as the prediction. Breiman [99] introduced bootstrap aggregating and random forests, where a given data sample is bootstrapped *M* times (i.e., randomly sampled with replacement). A tree-based model is then fit on a given bootstrap sample, and the final prediction the average of all bootstrap model predictions. Random forest and bootstrapped aggregated models can be efficient as the individual models are easily parallelizable.

An alternative approach to developing ensemble models is to build the final model sequentially with base or weak-learner models, where each model attempts to improve slightly over the previous aggregated models. Informally, in gradient boosting [100], simple models are added in a stage-wise manner optimized via gradient descent on the current model’s residuals:

4.5.4 $${Y}_{m}={Y}_{m-1}+\nu {y}_{m}$$

where *Y*_*m*_ is the gradient-boosted model at iteration *m*, *Y*_*m*−1_ is the previous iteration’s full model, *y*_*m*_ is the current base or weak-learner model, and *ν* is a penalization parameter between 0 and 1 that prevents the algorithm from proceeding too quickly and thus reducing effectiveness.

Ensemble models have been used with great success in geospatial exposure modeling. Random forest has been used in multiple studies to predict groundwater nitrate vulnerability [10, 101–103]. Ransom et al. [104] utilized boosted regression trees to predict groundwater nitrate concentrations in the Central Valley aquifer in California, USA. Gradient and extreme gradient boosting have also been used extensively to model spatiotemporal concentration of air pollutants such as PM_2.5_ [105–107]. Zhan et al. [108] added a spatial weighting to gradient boosting and reported better results than without geographic weighting for the spatiotemporal prediction of PM_2.5_. Lastly, new approaches have added Gaussian processes to random forest [109] and gradient boosting [109] to improve ensemble models with spatial data.

### Mechanistic or chemical transport

The exposure models discussed in previous sections are considered statistical models. Conversely, mechanistic or chemical-transport models (CTM) represent a class of models derived from basic principles of physics and chemistry, such as conservation of energy, resulting in a system of partial differential equations [110]. Many CTM used in exposure science are derived from a general advection-dispersion model based on the principle of conservation of mass [111, 112]:

4.6.1 $$Y(p):= \frac{\partial {C}_{i}}{\partial t}=-{{\boldsymbol{\nabla }}}\cdot \left({{\bf{v}}}{C}_{i}\right)+{{\boldsymbol{\nabla }}}\cdot \left(D{{\boldsymbol{\nabla }}}{C}_{i}\right)+{\sum }_{j=1}^{n}{r}_{i,j}+{G}_{i}$$

where *Y*(*p*), the exposure measure of interest at locations *p* = (*s*, *t*), is defined as ( ≔ ) *C*_*i*_, the concentration of species $i,{{\boldsymbol{\nabla }}}\cdot \left({{\bf{v}}}{C}_{i}\right)$ is the advective transport out of a defined domain due to a velocity such as wind or water flow, ${{\boldsymbol{\nabla }}}\cdot \left(D{{\boldsymbol{\nabla }}}{C}_{i}\right)$ is the diffusive transport with diffusion coefficient *D*, and ${\sum }_{j = 1}^{n}{r}_{i,j}$ is the net formation of species *i* from all of species *j*, and *G*_*i*_ is the net internal generation of pollutant species *i* such as emissions and deposition losses. ***∇***⋅ and ***∇*** are the spatial three-dimensional divergence and gradient operators, respectively. The general form of equation (4.6.1) provides a starting point for most CTM derivations. The exact derivation depends on factors such as the extent of the property, number of phases (e.g., gas, particle), the conservation law (e.g., mass, momentum), closure relations, and numerical approximations. For example, for air quality, the entire diffusion term is often ignored since it is minimal compared to advective transport. However, in groundwater transport, diffusive transport is typically an important term.

A large community of researchers are focused on elucidating the physical and chemical transport outside the human health context using CTM, but, their usage as a geospatial exposure assessment tool is also a common objective. In the air quality and health field, Community Multiscale Air Quality (CMAQ) model [113], Comprehensive Air Quality Model with Extensions (CAMx) [114], Weather Research and Forecasting model coupled with Chemistry (WRF-Chem) [115], and the Modern-Era Retrospective analysis for Research and Applications Version 2 (MERRA-2) [116] are examples of actively developed CTM. Since the CTM are large systems of partial differential equations, they are computational demanding and often require specialized training. To circumvent some of the computational issues and help geospatial exposure modeling scenarios, reduced complexity CTM have been developed. Tessum et al. [112] developed the Intervention Model for Air Pollution (InMAP), which utilizes variable grid sizes and simplified physics and chemistry to provide computationally scalability. Reduced complexity models excel in testing “what-if?” with exposure assessments by simulating with different parameterizations such as zeroing out emissions by location or industry. Tessum et al. [117] utilized InMAP to evaluate multiple exposure scenarios of air pollution emissions, industries, and the exposed racial and ethnic populations.

Dispersion models are a class of mechanistic models commonly used for air quality exposure assessments that focus on transport and neglect or simplify chemistry. For example, R-LINE is a steady-state Gaussian plume model designed to simulate line-type source emissions (e.g., mobile sources along roadways) by numerically integrating point source emissions [118]. AERMOD is a USEPA regulatory industrial and point source dispersion model. Dispersion models are excellent tools for geospatial exposure assessment for small-scale (i.e., city-block) applications when in-situ monitoring data is not available or when the focus is on a small set of point or line sources.

For groundwater assessment, the USGS developed and maintains MODFLOW, a three-dimensional mechanistic groundwater flow and transport model [119] comparable to CMAQ and MERRA-2 for air quality. Gallagher et al. [120] utilized MODFLOW to estimate the historical impact of effluent on drinking water wells for calculating exposures to examine the association between wastewater effluent in drinking water and breast cancer. Reduced-complexity mechanistically-based hydrological models are available that simplify transport or chemistry components of the full conservation equation. Beven and Kirkby [121] developed a topography-based hydrological model, a conceptual tool based on the topographic wetness index that allows the simulation of hydrological process, particularly the dynamics of surface or subsurface contributing areas, in a simplified mechanistic approach.

Since CTM have a high expertise and computational burden, many governmental agencies and consortia make model output across varying spatial and temporal domains available on-line. CMAQ-based output of air toxics are available for the conterminous US for 2014 through the National Air Toxics Assessment. The NASA Global Modeling and Assimilation Office provides regular MERRA-2 output, including daily and sub-daily simulations of many air quality parameters including ozone, aerosols, and gas-phase pollutants.

### Hybrid

The final class of geospatial exposure models brings together many aspects of the previous models into a hybrid framework. The fundamental principle of hybrid models is similar to the motivation of ensemble models in machine learning: a consensus of multiple models offers advantages in terms of robustness and ability to handle complex data. In geospatial exposure applications, hybrid development is difficult and time consuming since it requires fitting multiple types of models, but hybrid models consistently outperform single model methods in terms of prediction accuracy. The types of hybrid models used in exposure modeling include (1) model output as a direct input to another model, (2) sequential application of models on model residuals, and (3) ensemble models.

The first type of hybrid model involves using the output of one geospatial model for another geospatial model. A common implementation is the integration of proximity models (section “Proximity”), CTM (section “Mechanistic or chemical transport”), or satellite-derived data as covariates in models such as a GP [122] or ANN [92]. This is a straightforward approach to integrating models with varying spatial or temporal scales since there is no strict requirement that covariates scales match that of the outcome.

The second type of hybrid model is the sequential application of models on the previous model’s residuals. The most common example is a LUR (i.e., linear regression) followed by a geostatistical model such as GP/kriging [123, 124] or BME [125]. This two-stage approach uses geostatistical modeling of the residual correlation of the LUR model, which results in improved overall prediction accuracy. However, the two-stage approach is non-optimal since the LUR model assumes independent errors, effectively admitting that the LUR model assumption was violated. Consequences include inflated LUR coefficient variances, reduced model selection sensitivity and specificity, and overall reduction in prediction accuracy due to mis-specified models in the LUR stage. Messier and Katzfuss [33] developed an LUR-kriging approach that simultaneously selects and estimates LUR coefficients and GP covariance parameters with a scalable penalized likelihood approach. This approach to hybrid sequential model fitting is more optimal as evidenced by improved prediction and model selection.

The third type of hybrid model is the ensemble model approach, also known as meta-learners or super-learners [126]. In this approach, multiple geospatial models are fit, and the final prediction is then derived from those models, using, for example, the weighted average of all the models, or using another model with those models as inputs. Requia et al. [9], Danesh Yazdi et al. [127], and Yu et al. [128] fit multiple ML models followed by cross-validated meta-model weighting final ensemble predictions of air pollution. Murray et al. [129] utilized a Bayesian model averaging method that provides full uncertainty quantification in base and ensemble model predictions.

Hybrid models also serve multiple purposes when combining mechanistic models (e.g., CTM) and observation-based statistical models. First, for CTM, calibration to observations reduces known biases due to errors in emissions inventory, chemical mechanisms, and large spatial resolution. Second, hybrid models downscale, or increase spatial resolution of, CTM and satellite imagery while taking advantage of benefits such as detailed emissions, meteorology, and large spatial and temporal domains.

## Geospatial data integration

Geospatial data linkages are required for calculating geographic covariates and connecting exposure data and models to health data. To facilitate discussion of data linkages, some pre-requisite definitions are required.

*Geometry* refers to the spatial representation of objects including the shape, size, and relative position. There are three types of spatial geometry: point, line, and area (e.g., polygons, grids).

The *scale* of geospatial data refers to the extent at which a process varies. In other words, the minimal distinguishable size, length, or extent of spatial variability. Scale is both a spatial and a temporal property.

The *support* refers to the geometry, volume, shape, and orientation for geospatial data [130]. It is rare that multivariable exposure or health datasets are exactly aligned in their support, thus assumptions and methods are needed to ensure clarity and validity in linking geospatial data. Linking datasets may include transforming data to another support (e.g., point to area, area A to area B), which is known as the *change-of-support problem* [130, 131].

For non-uniform data, spatial geometries of point, line, and polygons are referred to as *vector* data. Vector data are defined by spatially referenced points (e.g., latitude/longitude pair) or sequences of spatially referenced points for lines and polygons. For uniform geometry data, the data may be represented as a grid where each grid cell represent an area value and is referred to as *raster* data. Raster data can be simply defined by a geographic bounding-box (i.e., 4 corners), coordinate reference system, and a grid cell size. Geospatial data can be converted from raster-to-vector or from vector-to-raster to facilitate linkages (section “Spatial linkage considerations”); however, these conversions can also introduce error to exposure assessment [8].

### Spatial linkage considerations

**Fig. 5:**
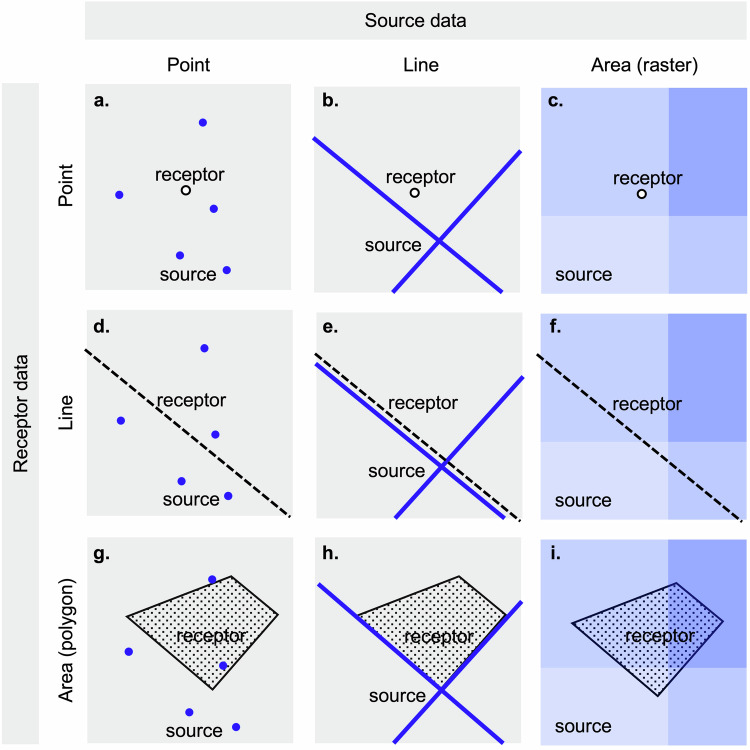
Illustration of exposure and health data by spatial geometry relationships. Point sources (e.g. industrial plants) can be linked via distance relationships and areal summarization to point receptor (e.g. home address), lines (e.g. roads) and areas (e.g. census tracts) as shown in **a**, **d**, **g**, respectively. Line sources impacting point, line, and area receptors are shown in **b**, **e**, **h**, respectively. Line receptors (i.e. **d**–**f**) are possible such as a range or uncertain addresses, but much less common than point and area receptors. **c** demonstrates how areal source data such as gridded satellite data can be assigned to point receptor locations. Note that repeat values are possible if multiple point receptors lie within the same source grid or polygon. **i** shows area-to-area statistics or summarizations.

Here, we describe spatial linkages based on the source and receptor concept. These linkages apply for exposure to health data connections, exposure to exposure transformations, and exposure metric calculations. Available linkage methods depend on the combination of spatial geometry data types, as illustrated in Fig. 5. Table 2 provides examples of source and receptor data for each spatial geometry type represented in Fig. 5.

**Table 2:** Example source and receptor data by spatial geometry type.

Geometry	Source	Receptor
Point	• Power plant stack as source of air pollution • Industrial facility wastewater outlet pipe as source of water pollution	• Patient geocoded home addresses • Fixed air pollution monitoring station
Line	• Street network as source of vehicle tailpipe air pollution emissions • Aircraft flight path as source of noise	• Travel path for health cohort participant • Route for mobile air pollution monitoring
Polygon	• Wildfire burn area as source of air pollution • Agricultural land use area as source of water pollution	• Population data for administrative or political boundaries (e.g., census tracts, postal codes) • Watershed boundary
Raster	• Gridded temperature data from satellite • Gridded air pollution predictions from mechanistic model	• Gridded population data

Figure 5a shows point to point connections. These connections are common for calculating exposure metrics at pollutant monitoring locations. Geographic covariates, such as the sum of exponentially decaying contribution, are calculated for the exposure metric of interest (e.g., fine particulate matter (PM_2.5_) air pollution concentration) at the receptor location with weighted contributions from the point source data. Point-to-point linkages are also possible for connecting point source exposure data to health receptor locations. For instance, the receptor data may be a patient residential location and the point source data may be exposure measurements at nearby monitors. An average of the point source data within a given circular buffer is a reasonable exposure metric. However, we recommend developing exposure models based on the point source data as opposed to observation statistics which may be biased due to differences in observation (point source in this case) density for each receptor.

Figure 5b shows the line source data contributing to a point receptor. This is most common in exposure geographic covariate calculations such as estimating the contributions of road emissions to a monitoring location [118]. The line is typically discretized into a sequence of points followed by point-to-point calculations. Similar to point-to-point connections, a line source exposure can be linked to point receptor health data; however, it is likely a crude proxy such as a proximity model (section “Proximity”). We recommend calculating a covariate that accounts for the line source data, then connecting that with the point receptor health data.

Figure 5c shows the areal (raster) source data contributing to a point receptor. This linkage is common for both exposure geographic covariate calculations and exposure to health receptor connections. Raster data sources are linked to point measurement data by either (1) extracting the exact grid cell value of the raster that intersects the point or (2) calculating a weighted average of nearby raster grid cells, which smooths spatial variability in the raster source data.

Figure 5d–f represents line geometry receptor data, which is shown for completeness; however, it is rare that exposure or health data are represented with this geometry.

Figure 5g, point source data to areal (polygon) receptor data, is a change-of-support problem that is commonly used in exposure data/models to health data connections and calculations of exposure geographic covariates. The point-level support is up-scaled to areal support by calculating the average of point-source data within the polygon receptor data. Equation (5.1.1) shows the calculation from point support, *s*, to areal support with area ∣*B*∣:

5.1.1 $$X(B)=\frac{1}{| B| }{\int}_{\!\!\!\!B}X(s)ds$$

Fig. 5h shows the connection between line source data and polygon areal receptor data. This linkage is common for linking exposure metrics directly to areal-level health data such as a disease rates within census boundaries. Areal statistics such as total line length and line density can be calculated for each receptor polygon. If a pollutant source is associated with the line source, then it is more appropriate to calculate an exposure covariate to a gridded receptor, then use areal source to areal receptor linkages.

Figure 5i shows the areal source to areal receptor linkage. This is a change-of-support problem that involves transforming the scale of the source data. Equation (5.1.2) shows this linkage is a weighted average of the source data, where the weights are the proportion/fraction, *p*_*i*_, of grid cell *i* in the receptor boundary *B*:

5.1.2 $$X(B)=\mathop{\sum }_{i=1}^{k(B)}{p}_{i}{X}_{i}$$

where there are *k*(*B*) total overlapping units in *B*.

### Temporal linkage considerations

Linking geospatial data can involve accounting for differences in temporal support. Temporal scale (i.e., frequency) can vary from seconds to decades among geospatial data sources. For example, meteorological data from satellites is often available at the hourly scale, whereas social data from surveys is often available at the annual or decadal scale. Similarly, the temporal range (i.e., time period covered) can vary from shorter-range– such as study-specific data collection campaigns (e.g., covering a single season)– to longer-range– such as routine data collection from government agencies (e.g., covering multiple decades).

Several approaches are available to link data with disparate temporal support. Finer-scale temporal data can be linked to coarser-scale temporal data using aggregate summary metrics (e.g., by calculating annual mean of hourly measurements). Likewise, coarser-scale temporal data can be linked to finer-scale temporal data using various temporal downscaling methods (e.g., statistical downscaling methods applied to meteorological data [132]). Data with disparate temporal coverage can be linked by nearest available time point or using temporal interpolation methods [133]. For example, in the limit, purely spatial covariates (e.g., built environment, social determinants of health) can be used with temporally varying covariates in models such that the spatial covariate value is repeated across time.

Additionally, there is growing interest in longer-range geospatial exposure models that can help characterize the exposome throughout the life course (e.g., covering multiple decades in the past as well as into the future). Considerations for linking data for such longer-range models include changes in data collection methods over time (e.g., in sensor technology or survey design), sparse availability of historic environmental data, and uncertain sustainability (i.e., future availability) of data sources.

### Special health data linkage considerations

Here, we review special considerations for linkages involving health data for individuals. Sources of health data for individuals include clinical data (e.g., patient electronic health records (EHR)), research data (e.g., health cohort participant data), and health insurance data (e.g., payer claims data). Geospatial exposure data– such as air quality, noise, and greenness data– is typically linked to health data for individuals by calculating exposure metrics that account for the specific spatial locations and time periods of exposure for each individual. Important considerations in calculating and analyzing these exposure metrics include preparing geospatial information for individuals, accounting for time-activity patterns in exposure metrics, protecting privacy of individuals, and interpreting uncertainty in exposure metrics.

Geospatial information for individuals is needed for linking health and geospatial exposure data. Home addresses– which are routinely collected by health data providers for administrative purposes at specific time-points– are a common source of geospatial information used for linkages. Geocoding– the process of translating addresses from text (e.g., street address format) to coordinates (i.e., latitude and longitude)– can be technically challenging. Available geocoding methods have varying spatial accuracy, match rates, automation, and privacy protection strategies [134, 135]. Addresses collected for administrative purposes may be missing information needed for geocoding (e.g., incomplete street number). Population-level geographic units, such as ZIP codes or counties, are also commonly used as geocodes for individuals; however, these represent coarser-scale spatial information. These are used when address information is not available, or when studying exposure-health relationships at coarser spatial scales, such as neighborhood-level social determinants of health for individuals.

Accounting for time-activity patterns, which describe how individuals move through time and space (e.g., from home to work and other locations), is an important consideration for calculating geospatial exposure metrics– particularly for exposures with higher spatial and/or temporal variability (e.g., traffic-related air pollution exposure) [136, 137]. Time-activity data can range in detail from current home address, to home and work address histories, to personal geolocation data (e.g., from wearable GPS). Several approaches are available to account for time-activity patterns in the exposure metrics used for linkages [138]. For example, a time-activity weighted average exposure metric can account for exposures at different point locations (e.g., home, school, and work addresses) by weighting exposures estimated at each location in proportion to the amount of time an individual spends at each location [139]. An activity space-based exposure metric can also account for exposures during travel between the different point locations by reflecting time-integrated average conditions within a spatial area enclosing different point locations and/or travel paths between locations [140]. Where fine-scale time-activity data as well as fine-scale geospatial exposure data are available, various time- and space- integrated average exposure metrics can account for exposures at each specific spatial location at each specific time-step (e.g., at 1-min intervals) [141].

Importantly, geospatial information for individuals (e.g., addresses, geocodes, time-activity patterns) is sensitive, potentially identifying information. Laws and institutional policies (e.g., Health Insurance Portability and Accountability Act (HIPAA) in the US [142]) require protecting individuals’ geospatial information. Thus, privacy protecting strategies are needed throughout the data processing pipelines used in geospatial exposure assessment. For example, many available geocoding tools require sharing addresses with geocoding companies over the internet– which risks exposing those addresses. Geocoding strategies for protecting privacy include using offline geocoding tools [143] and developing privacy-aware APIs for accessing online geocoding tools [144]. There are also privacy concerns associated with developing integrated datasets of geospatial exposures for individuals, due to re-identification risks. Approaches for reducing this risk include geographic masking approaches, such as applying spatial blurring (or, introducing noise) to geocoded addresses before linking individual exposure estimates [145].

Interpreting potential sources of error in exposure metrics is an important consideration in analysis of exposure and health outcomes. There are several potential sources of error in exposure metrics estimated from geospatial models compared to, for example, direct personal exposure measurements. These sources include geocoding errors (i.e., uncertainty in spatial location of address), exposure model errors (i.e., differences between model predictions and measurements), and other exposure misclassification errors (e.g., errors owing to limited time-activity information).

## Open-source tools

Linking geospatial exposure data with health data involves a range of geospatial data engineering challenges [143, 146–148]. Various open-source tools have been developed to support addressing these challenges. Supplementary Table A lists examples of available open-source tools, such as code, software, and web applications, and categorizes them by the specific steps they address in Fig. 2. Data sources and tools are rapidly evolving; Supplementary Table A represents a snapshot of the current landscape. Catalogs and repositories (such as the CHORDS catalog [21], NASA EarthData [149], CAFE repository [150], and others in Supplementary Table A) aim to provide continuously updated information about available data and tools.

Available tools include open-source geographic information system (GIS) software (e.g., QGIS [151]), R packages (e.g., sf [152]), and Python libraries (e.g., GeoPandas [153]) that broadly support geospatial data engineering and analysis. Other specialized tools help find open geospatial data (e.g., National Environmental Public Health Tracking Network data catalog [154]), access subsets of large geospatial datasets (e.g., OPeNDAP software [155]), integrate disparate data (e.g., GriddingMachine software [156]), develop geospatial exposure models (e.g., terra R package [157]), share data (e.g., NetCDF [158]), link addresses with geospatial exposure estimates (e.g., DeGAUSS software [159]), and calculate geospatial exposure metrics (e.g., hurricaneexposure R package [160]). Continued development of open-source tools (such as those in Supplementary Table A) can further reduce technical barriers to geospatial exposure assessment.

## Conclusion

Looking forward, technological advancements in ML algorithms and remote sensing data could dramatically improve the accuracy and spatial resolution of exposure models. For example, approaches have explored the integration of image recognition algorithms to calculate high-resolution geographic covariates [161]. The dizzying pace of advancements in large-language and computer vision models [162] offers exciting opportunities for transformative improvements in geospatial exposure assessment. An example near-term advancement is AI-assisted code development that reduces the modeling expertise burden. Nonetheless, these advancements must be made with domain knowledge in mind and with careful ethical considerations for their connections with health data. Moreover, efforts should be made to increase data sharing and language harmonization among fields that touch the geospatial realm, such as environmental science, epidemiology, toxicology, and public health. By addressing these challenges and leveraging the potential of geospatial exposure modeling, we can advance our understanding of the environmental determinants of health and promote evidence-based interventions to improve public health.

## Supplementary information

Supplementary Information
